# Functional status decline as a measure of adverse events in home health care: an observational study

**DOI:** 10.1186/1472-6963-6-162

**Published:** 2006-12-20

**Authors:** Tanya Pollack Scharpf, Natalie Colabianchi, Elizabeth A Madigan, Duncan Neuhauser, Timothy Peng, Penny H Feldman, John FP Bridges

**Affiliations:** 1 Case Western Reserve University, Department of Epidemiology and Biostatistics, 10900 Euclid Avenue, Cleveland, OH 44106, USA; 2 Case Western Reserve University, Frances Payne Bolton School of Nursing, 10900 Euclid Avenue, Cleveland, OH 44106, USA; 3 The Center for Home Care Policy and Research, Visiting Nurse Service of New York, 107 East 70^th ^Street, New York, New York 10021, USA; 4 Department of Tropical Hygiene and Public Health, University of Heidelberg – Medical School, Im Neuenheimer Feld 324, D-69120, Heidelberg, Germany

## Abstract

**Background:**

Research that examines the quality of home health care is complex because no gold standard exists for measuring adverse outcomes, and because the patient and clinician populations are highly heterogeneous. The objectives in this study are to develop models to predict functional decline for three indices of functional status as measures of adverse events in home health care and determine which index is most appropriate for risk-adjusting for future quality research.

**Methods:**

Data come from the Outcomes and Assessment Information Set (OASIS) from a large urban home health care agency and other agency data. Prognostic data yields 49,437 episodes, while follow-up data yields 47,684 episodes. We tested three indices defined as substantial decline in three or more (gt3_ADLs), two or more (gt2_ADLs), and one or more (gt1_ADLs) ADLs. Multivariate logistic regression determines the performance of the models for each index as measured by the c-statistic and Hosmer-Lemeshow chi square (χ^2^).

**Results:**

Frequencies for gt3_ADLs, gt2_ADLs, and gt1_ADLs are 212 (0.43%), 783 (1.58%), and 4,271 (8.64%) respectively. Follow-up results are comparable with frequencies of 218 (0.46%), 763 (1.60%), and 3,949 (8.28%) for each index. Gt3_ADLs does not produce valid models. The model for gt2_ADLs consistently yields a higher c-statistic compared to gt1_ADLs (0.754 vs. 0.679, respectively). Both indices' models yield non-significant Hosmer-Lemeshow chi square indicating reasonable model fit. Findings for gt2_ADLs and gt1_ADLs are consistent over time as indicated by follow-up data results.

**Conclusion:**

Gt2_ADLs yields the best models as indicated by a high c-statistic and a non-significant Hosmer-Lemeshow χ^2^, both of which exhibit exceptional consistency. We conclude that gt2_ADLs may be preferable in defining ADL adverse events in the context of home health care.

## Background

Research that examines the quality of home health care is complex because no gold standard exists for measuring adverse events, and because the patient and clinician populations are highly heterogeneous. In 1999, the Center for Medicare and Medicaid Services (CMS) instituted the use of the Outcomes and Assessment Information Set (OASIS) for all home health care agencies to be used for internal and external quality assessment. In October of 2000, OASIS measures also became the basis for case-mix adjustment in a new home health care prospective payment system. Shaughnessy et al. developed a comprehensive framework using a two-stage process for assessing the quality of home health care which separates the effects of natural disease progression from the effects of substandard care. The process and the measures they developed were used by CMS as the model on which to base home health care quality improvement [1,2]. As part of its outcomes based quality initiative, CMS identifies 13 potential adverse events for patients in home health care. Adverse events are considered to be both rare and potentially preventable through provision of appropriate skilled care. The adverse events cover four main areas: emergency care, serious unexpected events, unmet care needs, and declines in health or physical functioning [3].

Declines in health or physical function refer to changes in the ability to perform activities of daily living (ADL) and instrumental activities of daily living (IADL) [4]. Measures of ADLs include basic tasks like being able to feed, bathe, and groom oneself, while IADLs involve life skills such as cooking and cleaning. Some researchers have cited the measure of ADLs as the most important outcome for measurement for individuals receiving long term care and hence, home care [5]. Currently, CMS considers declining ADL function to be an adverse event if a home health patient experiences a "substantial decline in three or more ADLs", where substantial is considered a minimum two-unit decline from the start of home health care to final discharge, regardless of the time frame [1,2]. The five ADLs included in the index are grooming, toileting, bathing, transferring, and ambulation. OASIS is unique in that it does not have each skill measured on the same scale and some skills allow for use of medical devices for assistance while others do not include devices (see Table 1). CMS provides public reporting for changes in discrete functional status items through the Medicare website; these items are risk adjusted as part of the public reporting function. However, the adverse event reports are not risk adjusted, primarily because the intent is that these adverse events are to be investigated at the patient level to determine whether there are overall quality improvement measures to be taken by the agency.

In this paper, we evaluate three indices of ADL decline among home health patient episodes of care at a large metropolitan home health care agency. One index is calculated in a comparable fashion to the one currently used by CMS, described above. Based on empirical and theoretical considerations, two additional indices are developed. The main question is whether the CMS definition is the most appropriate ADL index for defining adverse events in the context of home health care. A secondary question is the degree to which the indices may be affected by floor effects that may lead to biased results [6].

## Methods

The data consist of episodes of patient care derived from two separate six-month periods, the first of which is used to derive the analysis sample, with the second providing a follow-up sample for evaluating consistency over time. The same six months from adjacent years are used to minimize any seasonal bias across samples. This study is based on deidentified data and as such was granted exemption from ethical review by Case Western Reserve University.

The start of any given episode of care is defined as the first recorded OASIS assessment during the sample time frame for start of care or resumption of care. Resumption of care occurs when patients are returning to home health care after a transfer to an institutional setting. The end of a given episode of care is defined as the first discharge OASIS assessment occurring on or before 60 days following the start of the episode; if no discharge has occurred by 60 days, the closest OASIS assessment available at or before 60 days is used. Following the implementation of the home health prospective payment system for Medicare home health, CMS required agencies to conduct OASIS assessments every 60 days for patients still in care. Thus, the maximum length for an individual episode is 60 days. Because the time frames for each sample is 6 months, patients may experience multiple episodes within a sample.

### Sample

**Figure 1:**
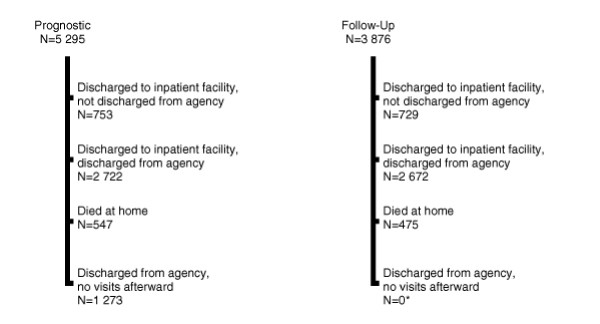
**Excluded Episodes from Prognostic and Follow-Up Datasets**. *CMS made coding changes in 2002 which accounts for this difference between the prognostic and follow-up datasets. Despite this difference, the datasets are similar with regard to included episodes.

There were a total of 54,732 and 51,560 patient episodes for the prognostic and follow-up datasets, respectively. Of these, 5,295 were excluded from the prognostic dataset and 3,876 were excluded from the follow-up dataset because they did not have outcome data (e.g. died, hospitalized) yielding a total of 49,437 and 47,684 episodes of care, respectively for the prognostic and follow-up datasets (see Figure 1).

### Dependent variables

As a first step to developing alternatives to the conservative CMS-based index, we evaluate two-unit change frequencies for each individual ADL (e.g., minimum two-unit declines in each category: grooming, toileting, bathing, transferring, and bathing). Incremental change for a one-unit decline for the five ADLs and for a three-unit decline for the five ADLs are also evaluated. These univariate analyses are done for two reasons:

• to determine which ADLs are driving the indices (i.e., comprising the largest frequency);

• to evaluate the frequencies of the magnitude of functional decline (e.g., to determine if most of the declines were one unit, two-unit, or three unit changes).

The univariate analyses provide additional information. The nature of home health care agencies allows for many nurses and practitioners to provide care. Therefore, a one-unit change is not considered enough of a difference since it may be attributable to different clinicians' views of dependency and hence may lead to more measurement error. While physical functioning skills are considered among the most reliable outcome measures in the OASIS [7-9], a one-unit change may be attributable to measurement error based on clinical judgment and the methods by which the data are coded (i.e. observation versus interview).

When evaluating declines in three or more ADLs exhibiting a three-unit change, the frequencies are extremely low (less than 0.20 percent). Declines in two or more ADLs exhibiting a three-unit decline are more frequent (more than 0.50 percent) but, still very small and incapable of providing meaningful statistical analyses. A three-unit decline is considered to be too large of a change, resulting in even fewer episodes experiencing ADL declines. Consequently, for all three indices, substantial indicates a minimum two-unit decline.

In addition to the incremental change issues aforementioned, another issue is the number of ADLs which need to have a substantial decline, where substantial is a minimum two-unit decline. One of the alternative indices is defined as substantial decline in two or more ADLs and is referred to as gt2_ADLs. A second alternative index is defined as substantial decline in one or more ADLs and is referred to as gt1_ADLs.

### Independent variables

There were 112 available covariates (including referent categories) between OASIS and other agency administrative data. These covariates fall into seventeen main categories. The number in parentheses indicates the number of covariates for each category. The constructs are: demographics (11), physician availability (2), financial factors (4), language (3), payment source (5), referral and personal health at baseline (16), pain assessment (4), integumentary status (3), respiratory status (1), sensory status (3), elimination status (5), housing and living arrangements (4), support and assistance in the home (8), neurological/behavioral/emotional status (5), medication assessment (7), ICD-9 primary diagnosis codes (22), ICD-9 primary diagnosis codes for the main diagnoses for this particular home health care agency (9). Physician availability is defined as the number of physicians available per 100,000 people per zip code and is divided into quintiles.

### Univariate analyses

Because it is unclear which of the five ADLs is most appropriate, it is not desirable to use an individual ADL outcome [10,11]. However, incremental changes in ADLs are evaluated to determine if any specific ADL is a major contributor for declines experienced in the indices. An additional concern when examining ADL outcomes is the number of cases excluded due to 'floor' effects. If an ADL as measured at the start of an episode is already at a level of functioning such that it is impossible for a decline to be identified by an index, it is dropped from consideration. However, this introduces a potential bias in excluding from the calculation of the respective indices those episodes beginning with greater severity in ADL functioning. Thus, gt3_ADLs can only be calculated for episodes that begin with the possibility of worsening in three of the five ADLs; therefore, episodes at the 'floor' for gt3_ADLs are those unable to worsen in three or more ADLs. Likewise, for gt2_ADLs, 'floor' episodes are unable to worsen in at least four ADLs; and 'floor' episodes for gt1_ADLs are unable to decline in any of the ADLs.

### Multivariate analyses

We estimate a series of multivariate logistic regression models using SAS version 8.0 [12]. Variables included in the initial models are selected among the full set of independent measures, using three criteria: 1) a minimum of 5% frequency criterion; 2) risk factors with a correlation of 0.80 or higher presumably measure the same item [13] and when this occurs, one of the variables is randomly eliminated; and 3) bivariate analyses including the weighted baseline ADL status [14] due to its well documented association with functional decline [15-17], with p < 0.10 used as the selection criterion because the association between functional decline and many risk factors is unclear. Baseline ADL scores are weighted by dividing the score for each individual ADL by the total possible and then the five weighted scores are summed [18]. In the final step, forward stepwise multivariate logistic regression is configured to use a selection criterion of p < 0.05 [19].

### Analytical measures

For the multivariate logistic regression analyses, the c-statistic and the Hosmer-Lemeshow Goodness of Fit test are compared for each model. The c-statistic, calculated as the area under the Receiver Operating Characteristic (ROC) curve, represents the concordance between predicted probabilities and observed outcomes for all possible pairs of patients [20,21]. A c-statistic of 1.0 indicates perfect predictive discrimination while a value of 0.50 indicates the model performs no better than chance alone. A c-statistic over 0.70 is considered acceptable while values higher than 0.80 are considered excellent [22].

The Hosmer-Lemeshow Goodness of Fit test examines model calibration across a range of predicted probabilities [13], producing a single summary measure of the match between predicted and actual outcomes within deciles of the data. Within each decile, deviations between observed and expected number of declines in functional status are observed and expected numbers of episodes not experiencing functional decline are measured. In order to determine whether these deviations are larger than expected, these deviations are summed over the ten groups and compared to a χ^2 ^with 8 degrees of freedom [22]. For the Hosmer-Lemeshow test, a p-value that is not statistically significant is indicative of reasonable model fit. Consistency over time is assessed by comparing the models' results from the prognostic and follow-up datasets.

## Results

For the prognostic data, the population of episodes averages 71 years of age with 68% being female. The population is highly diverse: 36% white, 26% black, 26% Hispanic, 5% Asian, and 7% some other ethnicity. Clinical diagnoses cover a wide spectrum of conditions, with the most common being dyspnea (48%), circulatory diseases (28%), and skin lesions (24%). In general, compared to the entire dataset, the subsets of episodes experiencing substantial functional decline tend to have a larger proportion of the following: women, older patients, Black patients, low physician availability, low income, more comorbidities, and episodes covered by both Medicare and Medicaid health insurance. The follow-up data has similar demographic characteristics.

Gt3_ADLs, defined as substantial decline in three or more ADLs, consistently yields the lowest frequencies with results of 212 and 218 respectively for the prognostic and follow-up datasets (see Table 2). This conservative index only accounts for half a percent of either dataset. Gt2_ADLs, defined as substantial decline in two or more ADLs and gt1_ADLs, defined as substantial decline in one or more ADLs, yields substantially more frequent occurrences of decline. Gt2_ADLs yields frequencies of 783 and 763 respectively for the prognostic and follow-up datasets, accounting for about 2% of all episodes. Gt1_ADLs yields frequencies of 4,271 and 3,949 respectively for the prognostic and follow-up datasets, accounting for nearly 9% of all the episodes. All three indices exhibit stability with comparable frequencies in the prognostic and follow-up datasets.

We conclude that we have no 'floor' effects for gt1_ADLs or gt2_ADLs because the results are comparable with and without 'floor' covariates. Interaction is assessed and determined not to be present. Gt3_ADLs models do not converge, resulting in spurious results that are not shown.

The model for gt2_ADLs yields a c-statistic of 0.754 for the prognostic dataset; this is consistent with a c-statistic of 0.744 for the follow-up dataset (see Table 3). This represents only a 1% difference in the model's predictive ability. The Hosmer-Lemeshow χ^2 ^is 10.370 with a p-value of 0.240, indicating reasonable model fit. These results are supported with the follow-up data (χ^2 ^statistic with 8 degrees of freedom was 9.437 with a p-value of 0.307). The models for gt2_ADLs have 24 and 19 significant covariates for the prognostic and follow-up datasets, respectively.

The c-statistics from models of gt1_ADLs are substantially lower at 0.679 and 0.683 for the prognostic and follow-up datasets, respectively (see Table 3). The Hosmer-Lemeshow χ^2 ^is 9.781 with a p-value of 0.281, indicating reasonable model fit. The follow-up data results are comparable (χ^2^statistic with 8 degrees of freedom is 10.090 with a p-value of 0.259). The models for gt1_ADLs have 32 and 26 significant covariates for the prognostic and follow-up datasets, respectively.

Table 4 shows the regression results for gt2_ADLs and gt1_ADLs for both datasets. While some inconsistencies are evident among covariates that are significant in the prognostic dataset and not the follow-up dataset, all of the associations are consistent (i.e., covariates positively associated with declines in the prognostic data are also positively associated with declines in the follow-up data; negatively associated covariates for prognostic are also negatively associated for follow-up). Covariates that yield the strongest associations (positive or negative) are consistent across time periods (e.g., lowest physician availability, elimination status problems; daily pain, dyspnea, and surgical wounds).

## Discussion

Across the prognostic and follow-up datasets, simple frequencies of ADL adverse events were similar for all three indices. All three indices are most influenced by the substantial decline in the ADL for bathing. Declines in bathing may be most frequent due to the difficulty of independent use of showers and bathtubs for elderly, infirm patients.

Gt3_ADLs, which is based on the current CMS definition of an ADL adverse event, was too conservative, yielding few episodes experiencing functional decline and numerous 'floor' episodes as compared to the alternative indices. However, there are also benefits to using gt3_ADLs. Continuing to use the implementation of the CMS defined ADL index promotes consistency in process of care investigations across the home health care industry. Furthermore, this index is infrequent enough to be considered rare, a caveat of being an adverse event. However, when events are very rare, determining the root cause of problems is difficult. Likewise, it is difficult to evaluate if implemented solutions have been effective with rare events.

The proposed indices gt1_ADLs and gt2_ADLs offer advantages of their own. The gt1_ADLs models adhere to the '×10' guideline and is consistent with the high part of the range (4–8%) often found in the hospital literature [23-26]. However, gt1_ADLs may be too frequent to be considered rare, a necessary attribute of adverse events with ~10% of patients experiencing such an event. Gt2_ADLs adheres to the '×10 rule' [27], a statistical guideline advising a dichotomous outcome to be, at minimum, ten times as large as the number of covariates being analyzed. The entire dataset has 112 covariates; prognostic and follow-up data result in 783 and 763 episodes respectively, just slightly under this recommendation.

There may be several important policy and clinical implications of implementing the use of gt2_ADLs as an ADL index for defining adverse events in the context of home health care. While still considered rare (less than 2% of the entire population), gt2_ADLs is more frequent and by definition, less restrictive. Requiring the decline of only two ADLs compared to three (e.g. gt3_ADLs), gt2_ADLs encompasses both a larger frequency of episodes and a population experiencing slightly less functional decline and hence, one less likely to have episodes at the "floor" at baseline. A slightly larger and less incapacitated population may provide more valid results for both case-mix and for evaluating changes in clinical and/or policy practices.

### Limitations

The OASIS dataset utilized in this study represents the population of episodes for the largest not-for-profit home health care agency in the United States, but is not necessarily representative of the vast majority of home health care agencies nationwide. For this reason, these results are not generalizable to other home health care agencies. As well, the size of this one agency, as compared to smaller agencies, may mean that our results are more stable in comparison to smaller agencies. A second limitation is the use of a different calculation from CMS in developing the indices. The CMS definition does not include any time restriction in comparison to our 60-day limit. We would anticipate our definition to capture declines in functional status more quickly as episodes are reassessed in shorter time periods. Therefore, we may be increasing the number of episodes considered "floors".

Another limitation is the way in which the information is compiled; the datasets used are unable to be linked, making it impossible to know whether patients in the prognostic data are also in the follow-up data. The assumption was that these are independent samples for analytical purposes. Furthermore, patients within each dataset may have been represented more than one time. The unit of analysis was episode of care defined as a maximum of sixty days. This could bias results as patient health characteristics could be correlated from one episode to another [28]. However, 67% of the patients were on their first episode. There was an average 1.5 episodes per patient making correlated episodes an unlikely source of bias.

From an analytical standpoint, it may have been advantageous to use a multinomial model to deal with the issue of competing risks (i.e. more episodes were excluded due to admittance to a hospital or other inpatient facility than experienced the functional decline). However, a multi-nomial model was developed and failed to converge.

## Conclusion

The models for gt2_ADLs provide the most valid index for predicting patients that will experience functional decline. This conclusion is based on criteria of c-statistics above 0.70; non-significant Hosmer-Lemeshow χ^2 ^indicating reasonable model fit, and the requirement of fewer covariates to produce valid models indicative of greater efficiency as compared to gt1_ADLs. These results are proven reliable with comparable follow-up data results.

The use of gt2_ADLs for defining ADL adverse events in home health care results in a slightly larger and less incapacitated population which may be more appropriate for case-mix adjustment and evaluating changes implemented in clinical and/or policy practices. However, this study provides only a first step in evaluating these adverse event indicators. Further research is necessary to assess the ability of these indices to be used in case-mix adjustment for evaluating quality in home health care. As well, the larger question of the quality of care in home health care using these measures remains unanswered–comparisons of these functional status measures with other external appraisals of quality would contribute valuable information in home health care quality research.

## Competing interests

The author(s) declare that they have no competing interests.

## Authors' contributions

TPS conceived the study, carried out the data analyses, and drafted the manuscript.

NC participated in the design and coordination of the study.

EAM participated in the design of the study and helped to draft the manuscript.

DN participated in the design and coordination of the study.

TP assisted in the data analyses and revising the manuscript critically for important intellectual content.

PHF contributed with the acquisition of data and revising the manuscript.

JFPB helped conceive the study and participated in its design, analysis, and manuscript revisions.

All authors read and approved the final manuscript.

## Pre-publication history

The pre-publication history for this paper can be accessed here:



## Figures and Tables

**Table 1:** Definition of Individual ADLs and ADL Scales on OASIS

ADL	Definition of Values
ADL: Grooming*	0 = Able to groom self unaided, with or without use of assistive devices
	1 = Grooming utensils must be placed within reach
	2 = Someone must assist the patient to groom self
	3 = Patient depends entirely upon someone else
ADL: Ability to dress upper body	0 = Able to obtain, put on, and remove clothing
	1 = Able to dress upper body without assistance
	2 = Someone must help patient
	3 = Patient depends entirely on someone else
ADL: Ability to dress lower body	0 = Able to obtain, put on and remove clothing
	1 = Able to dress lower body without assistance
	2 = Someone must help the patient
	3 = Patient depends entirely on someone else
ADL: Bathing*	0 = Able to bathe self in shower or tub independently
	1 = With the use of devices, is able to bathe
	2 = Able to bathe with the assistance of a person
	3 = Participates in bathing in shower or tub
	4 = Unable to use the shower or tub, but able to assist
	5 = Unable to effectively participate in bathing, totally bathed by another person
ADL: Toileting*	0 = Able to get to and from the toilet independently
	1 = When reminded assisted or supervised
	2 = Unable to get to and from toilet; can use bedside commode
	3 = Unable to get to from the toilet or bedside commode; can use a bedpan
	4 = Is totally dependent for toileting
ADL: Transferring*	0 = Able to independently transfer
	1 = Transfers with minimal human assistance
	2 = Unable to transfer self but is able to bear weight
	3 = Unable to transfer self and is unable to bear weight
	4 = Bedfast, unable to transfer but is able to turn
	5 = Bedfast, unable to transfer and is unable to turn
ADL: Ambulation/Locomotion*	0 = Able to independently walk
	1 = Requires use of a device
	2 = Able to walk only with the supervision
	3 = Chairfast, unable to ambulate but can use wheelchair
	4 = Chairfast, unable to ambulate and or use wheelchair
	5 = Bedfast
ADL: Feeding or eating	0 = Able to independently feed self
	1 = Able to feed self but requires help in setup
	2 = Unable to feed self and requires human assistance
	3 = Able to take in nutrients orally and tube fed
	4 = Unable to take in nutrients orally; tube fed
	5 = Unable to take in nutrients orally or by tube feeding

*Indicates this ADL was included in all three of the ADL indices.

**Table 2:** Descriptive Statistics for Individual ADLs, ADL Indices, and 'Floors'

	Prognostic Frequency	Prognostic 'Floors'	Prognostic Total n*	Follow-Up Frequency	Follow-Up 'Floors'	Follow-Up Total n*
Outcome	n (%)	n (%)	n	n (%)	n (%)	n
Gt3_ADLs	212 (0.47%)	4 657 (9.4%)	44780	218 (0.50%)	4 217 (8.8%)	43467
Gt2_ADLs	783 (1.69%)	3 203 (6.5%)	46234	763 (1.71%)	2 968 (6.2%)	44716
Gt1_ADLs	4 271 (8.98%)	1 896 (3.8%)	47541	3 949 (8.60%)	1 757 (3.7%)	45927
Grooming	904 (2.74%)	16 482 (33.3%)	32955	867 (2.70%)	15 577 (32.7%)	32107
Bathing	2 133 (4.81%)	7 802 (15.8%)	44307	1 939 (4.78%)	7 093 (14.9%)	40591
Toileting	911 (2.19%)	5 130 (10.4%)	41635	842 (1.96%)	4 700 (9.9%)	42984
Transferring	728 (1.54%)	2 138 (4.3%)	47299	708 (1.55%)	2 024 (4.2%)	45660
Ambulation	675 (1.48%)	3 933 (8.0%)	45504	652 (1.48%)	3 569 (7.5%)	44115

*n varies for each outcome since the number of episodes at the 'floor' varies.

**Table 3:** Summary of Analytical Measures

	Prognostic Models		Follow-Up Models	
Measure	Gt2_ADLs	Gt1_ADLs	Gt2_ADLs	Gt1_ADLs

c-statistic	0.754	0.679	0.744	0.683
# significant covariates	24	32	19	26
H-L Χ^2 ^(8 d.f.)*	10.370	9.781	9.437	10.090
p-value	0.240	0.281	0.307	0.259

*Hosmer-Lemeshow chi square with 8 degrees of freedom.

**Table 4:** Prognostic and Follow-Up Model Results for Gt2_ADLs and Gt1_ADLs

	Prognostic Models		Follow-Up Models	
**Parameter**	Gt2_ADLs	Gt1_ADLs	Gt2_ADLs	Gt1_ADLs
Only Significant Odds Ratios (OR) Reported	OR	OR	OR	OR
**Demographics**				
Female				
Age 75 to 84			0.84	
Age 85 and over	1.34	1.12		1.28
White				
Non-White				1.11
**Physician Availability**				
Highest quintile of availability	0.73			
Lowest quintile of availability	1.30	1.17	1.30	1.18
**Financial Factors**				
Highest quintile of income				
Lowest quintile of income	1.26	1.24		1.13
Financial Difficulties				
**Language**				
Spanish speaking only				
Translator needed		0.87		
**Payment Source**				
Medicaid				
Medicare and Medicaid				
Private or other payer	0.62	0.73		0.69
Managed care			0.65	
**Living Arrangements**				
Lives alone	0.82		0.84	
Lives with spouse				
Lives with Family		1.10		1.12
**Support/Assistance**				
Assistance, none	1.41	1.20		
Assistance in place of residence				
Assistance, out of residence				
Caregiver is spouse				
**Pain Status**				
pain, daily	0.76	0.84	0.82	0.87
Intractable pain				
**Referral/Personal Health**				
comorbidities (mean)		1.06	1.08	
Discharged from hospital within two weeks prior to admittance	0.75	0.80		0.80
Discharged from other facility within two weeks prior		0.82		0.80
Referral from a public hospital	1.43	1.18		
Life expectancy	2.02	1.42		
Medical regimen change within two weeks prior		0.89	0.69	0.87
Obesity			0.73	
Primary Diagnosis Severity				
**Referral/Personal Health**				
Poor prognosis	1.78	1.24		1.30
Poor rehabilitation		1.13	1.37	
High Risk Factor Summary Score				
Smoking				
Weighted ADL Summary Score	1.25	1.07	1.18	1.06
**Sensory Status**				
Vision impairment		1.13		
Hearing impairment	0.66	0.85		0.87
Speech impairment		1.39	1.46	1.39
**Elimination Status**				
Urine incontinence	1.86	1.46	1.87	1.45
Urinary catheter	2.80	1.86	1.73	1.69
Bowel incontinence	1.63	1.33	1.83	1.65
Incontinence				
**Neurological/emotional**				
Cognitive impairment, mild				
Cognitive impairment, high				
Confusion				
Depressed				
**Medication Assessment**				
unique number of drugs (mean)				
number of drug classes (mean)				
Psychotropic drug usage				
Caregiver knowledgeable of Medications		1.13		1.12
**ICD9 Codes of Primary Diagnosis**				
Circulatory without COPD				
Dyspnea	0.85	0.86	0.79	0.89
Injury				0.79
Musculoskeletal	0.71	0.77		0.82
Neoplasm	1.47	1.29	1.92	1.26
nervous system diseases	1.67	1.38	1.38	1.34
Respiratory diseases				
skin diseases	1.38	1.39		
Skin lesion				1.15
Surgical wound	0.70	0.71	0.62	0.67
**Among top ICD9 codes for agency**				
Congestive Heart Failure		1.25		
Pressure Ulcer	1.56	1.28	2.19	1.50

OR stands for Odds Ratio

All odds ratios reported were significant at p < .05
